# Novel Interaction between Proliferating Cell Nuclear Antigen and HLA I on the Surface of Tumor Cells Inhibits NK Cell Function through NKp44

**DOI:** 10.1371/journal.pone.0059552

**Published:** 2013-03-19

**Authors:** Nathan C. Horton, Stephen O. Mathew, Porunelloor A. Mathew

**Affiliations:** Department of Molecular Biology and Immunology and Institute for Cancer Research, University of North Texas Health Science Center, Fort Worth, Texas, United States of America; University of Sydney, Australia

## Abstract

NK cell function is closely regulated by numerous inhibitory and activating receptors binding corresponding ligands on the surface of target cells, providing vital first line defenses against infections and cancer. NKp44, originally discovered as an activating NK cell receptor, was recently found to elicit inhibitory effects on NK cell effector function through recognition of cell surface PCNA. Other reports have pointed to potential associations between NKp44 and HLA I molecules, as well as HLA I and Damage Associated Molecular Pattern molecules (DAMPs) on the surface of tumor cells. In this report, we have identified novel interaction between HLA I and PCNA on the surface of human tumor cells by confocal microscopy and immunoprecipitation. In addition to previous reports, we show PCNA on the cell surface where novel association with HLA I does not require the presence of NKp44 expressing NK cells and occurs with endogenous PCNA. The association of HLA I and PCNA forms the inhibitory ligand for NKp44, resulting in inhibition of NK cell cytotoxicity. We further postulate NCR ligands are composed of DAMP molecules localized to the cell surface, colocalizing with HLA I, and potentially heparin sulfate proteoglycans.

## Introduction

NK cells are a specialized population of lymphocytes of the innate immune system that defend against cancer as well as viral and microbial infections [1], [2]. NK cell activation, proliferation, and effector functions are regulated by the balance of signals delivered through activating and inhibitory receptors at the NK cell surface binding ligands on the surface of target cells [3]. Therefore, when a target cell over expresses activating ligands or ligands for multiple activating receptors, NK cells eliminate the target even if inhibitory receptors are engaged. Inhibitory receptors traditionally bind Class I Human Leukocyte Antigen (HLA I) molecules and signal through domains known as Immunoreceptor Tyrosine-based Inhibitory Motifs (ITIMS) while activating receptors bind other ligands and signal through Immunoreceptor Tyrosine-based Activating Motifs (ITAMS) or associate with adaptor molecules containing ITAMs [3], [4]. Among the activating receptors is a specialized class known as the Natural Cytotoxicity Receptors (NCRs), which include NKp30, NKp46, and NKp44 [5]. NCR ligand expression appears to be induced under pathological conditions; however, majority of the NCR ligands remain unknown and represent a vital area of current NK cell research [6].

NKp44 is a transmembrane glycoprotein of the Immunoglobulin super family encoded on chromosome 6 [7]. Originally reported as an activating receptor, NKp44 is now known to have dual functions conveying either activating or inhibitory signals, potentially through binding separate ligands [8], [9]. Surface expression and activating signaling through NKp44 requires the ITAM bearing accessory molecule DAP12 [9]. Currently, the identity of a ligand inducing activation signaling through NKp44 is still unknown. However, its activating ligand is over expressed in numerous tumor cell lines and induced in CD4 T cells by the gp41 envelope protein of HIV [10]–[12]. Inhibitory signaling through NKp44 occurs when the receptor engages cell surface Proliferating Cell Nuclear Antigen (PCNA), transducing signals through the ITIM located in the cytoplasmic tail of NKp44 [8]. PCNA performs a wide array of functions in the nucleus, particularly with DNA replication, repair, and maintenance [13]. Expression of PCNA is typically restricted to replicating cells; however, over expression of PCNA is often associated with cancer formation and progression, but also normal development in the deciduas of pregnant women, contributing to NK cell tolerance [13]–[16]. Therefore, cancer cells can simply abuse this unique form of tolerance mediated via NKp44 to survive and escape NK cell killing. Since NKp44 expression is restricted to only activated NK cells in peripheral blood, NKp44 plays a critical decision making role in regards to NK cell effector functions, depending on the nature of NKp44 ligands on the target cell surface [7], [9]. This not only makes NK cell modulation via NKp44 an attractive potential immunotherapy of the future, but also amplifies the importance of elucidating NKp44 ligand identities.

In the search to identify a ligand for NKp44, several key pieces of evidence have led us to investigate the possibility of HLA I playing a role in ligand formation. Betser-Cohen *et al.* recently found HLA I proteins coimmunoprecipitate with anti-NKp44 antibodies; reciprocally, NKp44 coimmunoprecipitates with anti-β-2-microglobulin antibodies [17]. Additionally, the Nef protein of HIV prevents surface expression of the activating NKp44 ligand on CD4 infected T cells, which is also consistent with the ability of Nef to retain HLA I intracellularly [18], [19]. Finally, Human Leukocyte Antigen-B associated Transcript 3 (Bat3), typically found in the nucleus, colocalizes with HLA I on the cell membrane of dendritic cells and tumor cells after nonlethal heat shock where it binds NKp30, activating NK cell effector functions [20], [21]. These studies suggest NCR ligands may be composed of a complex of HLA I and membrane proteins usually only found intracellularly, termed damage associated molecular pattern molecules (DAMPs), such as BAT3 and PCNA. These reports have led us to the hypothesis that HLA I on the surface of target cells participates in NKp44 mediated recognition by NK cells. In this study, we have identified interactions between HLA I and PCNA on the extracellular membrane of tumor cell lines, where HLA I and PCNA form a complex ligand recognized by NKp44, resulting in inhibition of NK cell cytotoxic function.

## Materials and Methods

### Cell Lines

Human diffuse B cell lymphoma cell line, DB (ATCC CRL-2289) [22], and DU145 (ATCC HTB-81) [23], were maintained in RPMI 1640 supplemented with 10% FetalPlex (Gemini Bio-Products, West Sacramento, CA), 2 mM glutamine, 10 mM HEPES, 10 mM Sodium Pyruvate, 10 mM non essential amino acids, and antibiotic-antimycotic solution containing penicillin G, streptomycin sulfate, and amphotericin B (Life Technologies, Carlsbad, CA). HEK-293 (ATCC CRL-1573) [24] and MCF-7 (ATCC HTB-22) [25] cells were grown in RPMI 1640 supplemented as above, except with 10% Fetal Bovine Serum (Atlanta Biologicals, Lawrenceville, GA). NK92-MI (ATCC CRL-2408), human NK cell line constitutively expressing IL-2, was maintained in Minimum Essential Medium Alpha Medium (Life Technologies, Carlsbad, CA) supplemented with 15% Fetal Bovine Serum, 0.2 mM inositol, 0.1 mM 2-mercaptoethanol, and 0.02 mM folic acid [26]. All cells were cultured at 37°C in a humidified 5% CO_2_/95% air environment.

### Construction and Expression of Soluble NKp44-Ig Fusion Protein

Soluble NKp44-Ig fusion protein was produced by fusing the extracellular domain of NKp44 with the Fc portion of human IgG. The extracellular domain of NKp44 was amplified by PCR (forward primer NKp44*Nhe*IFP-5′ TCGCTAGCGCAATCCAAGGCTCAGGT-3′ and reverse primer NKp44*BamH*IRP-5′ CTCGGGATCCGTGTCTGCAGG GCCA-3′). The amplified product was subcloned in front of human Fc gene at *Nhe* I and *BamH* I cloning sites in pCD5 vector, which contained the CH2 and CH3 regions of the human IgG1. Soluble NKp44-Ig fusion protein was produced by transiently transfecting the plasmid into HEK-293 cells using Fugene-6 transfection reagent (Roche Diagnostic Corporation, Indianapolis, IN). Cells were cultured in Optimem I (Life Sciences, Carlsbad, CA) reduced serum free media during transfection and collection of supernatants. Supernatants collected on days 2 and 3 after transfection were centrifuged to remove cellular debris, and then concentrated to 1 µg/ul with a 35,000 molecular weight centrifugal concentrator. Concentrated supernatants were verified for the presence of functional fusion protein by flow cytometry and western blotting. Supernatants of untransfected HEK-293 cells cultivated in Optimem I media were concentrated and used as negative controls in flow cytometry utilizing NKp44 fusion protein. A dose response curve was generated to determine optimum binding of NKp44-Ig prior to fusion protein use, which determined 70 µg of NKp44-Ig at a concentration of 1 µg/ul produces the largest shift of peak fluorescence without oversaturation.

### Flow Cytometry

DB cells were first incubated with Human IgG Fc fragment (Rockland, Gilbertsville, PA) to block interactions with fusion protein and Fc receptors. Cells were incubated with 2.5 µg of W6/32 anti-HLA I antibody (0.5 mg/ml) (Biolegend, San Diego, CA) or mouse IgG2a isotype control (0.5 mg/ml) (Invitrogen, Camarillo, CA) for 30 minutes and detected with 2.5 µg anti-mIgG (H+L)-PE (0.5 mg/ml) (Beckman Coulter, Brea, CA) to determine HLA I expression. To determine NKp44 ligand expression, DB cells were incubated with 70 µg of NKp44-Ig (1 µg/ul) fusion protein for 45 minutes followed by 2.5 µg of anti-hIgG-Fc-PE (0.5 mg/ml) (Beckman Coulter, Brea, CA) for 30 minutes. To block NKp44-Ig binding, cells were first incubated with 2.5 µg of anti-HLA I. Cells were then incubated with 70 µg of NKp44-Ig fusion protein for 45 minutes followed by 2.5 µg of anti-hIgG-Fc-PE for 30 minutes. As negative controls, cells were incubated with 2.5 µg of mouse IgG2a-FITC (0.5 mg/ml) isotype for HLA I expression or concentrated untransfected HEK293 supernatant and anti-hIgG-Fc-PE for NKp44 ligand expression. DB cells were incubated with 2.5 µg of anti-PCNA-Alexa Fluor 488 (0.5 mg/ml) (Biolegend, San Diego, CA) versus mIgG2a isotype to establish cell surface expression of PCNA. Cells were analyzed on a Beckman Coulter Cytomics FC 500 Flow Cytometer.

### Live Imaging Confocal Microscopy

DB and MCF-7 cells were incubated with 2.5 µg of W6/32 anti-HLA I antibody followed by 2.5 µg anti-mouse IgG2a-Dylight 594 (0.5mg/ml) (Biolegend, San Diego, Ca). Cells were then stained with 2.5 µg of anti-PCNA-Alexa Fluor 488 (0.5 mg/ml) and imaged on the Zeiss LSM 510 Confocal Laser Microscope using 40×, 1.2 NA, 0.28 WD (water), C-Apochromat objective utilizing 561 nm and 488 nm wavelengths at 43% and 57% transmittance respectively.

### Coimmunoprecipitation of PCNA with anti-β-2-microglobulin Antibody

DB and DU145 cells were lysed utilizing a buffer consisting of 0.5% nonidet P-40, 50 mM Tris-HCl pH 7.6, and 5 mM MgCl_2_. Immunoprecipitations were performed using Catch and Release v2.0 (Millipore, Billerica, MA) according to manufacturer’s specifications. DB cells express endogenous immunoglobulins and DU145 is reported to express cancerous immunoglobulins [27]. Therefore, to prevent cross reaction and cluttering when detecting PCNA through western blotting, endogenous and cancerous immunoglobulins were removed by incubating cell lysate with affinity capture ligand prior to immunoprecipitation with antiβ-2-microglobulin. Affinity capture ligand is provided in the immunoprecipitation kit and links the Fc portion of immunoglobulins to resin within a spin column. The pass through contains cell lysate removed of immunoglobulins derived from DB and DU145 cells. Pass through, termed precleared lysate, was analyzed by western blot to ensure removal of immunoglobulins and then used for immunoprecipitation. 500 µg of precleared cell lysate was mixed with buffer, affinity capture ligand, and 3 µg of either antibody against β-2-microglobulin (0.5 mg/ml) (BD, San Jose, CA) or mouse IgM isotype (0.5 mg/ml) (BD, San Jose, CA) and incubated in spin columns at 4°C for 1 hour. Elutions were performed with native buffer. Samples were denatured with 1% DTT, heated for 1 hour at 50°C to ensure dissociation of membrane protein aggregates and resolved on a 10% SDS-PAGE gel. After transfer to nitrocellulose, membranes were probed with anti-PCNA (1∶2,500) for 1 hour and anti-mIgG2a-HRP for 45 minutes (1∶2000) (Biolegend, San Diego, CA). Chemiluminescent imaging was performed with Immobilin (Milipore, Billerica, MA).

### ^51^Cr Release Assay

DB cells and NK92-MI cells were incubated with Human IgG Fc fragment to block interactions with Fc receptors on both cells prior to use. DB cells were labeled with ^51^Cr for 1 hr at 37°C and then incubated with either 15 µg of NKp44-Ig or 1 µg anti-PCNA (0.5 mg/ml), anti-HLA I (0.5 mg/ml), or mIgG2a isotype (0.5 mg/ml). Cells were then incubated with NK92-MI at ratios of 25∶1, 5∶1, and 1∶1 for 4 hours at 37°C. NK92-MI cells were previously confirmed to express NKp44 after two weeks of culture. Percent specific lysis was compared to DB cells incubated with mIgG2a isotype antibody or with no antibody as a positive control (No Blocking) of cell lysis under unblocked conditions. Alternatively, NKp44 was blocked on NK92-MI cells with 2.5 µg of anti-NKp44 antibody (0.5 mg/ml) (Biolegend, San Diego, CA) or 2.5 µg mIgG1 (0.5 mg/ml) isotype control antibody prior to incubation with DB cells incubated with mIgG2a isotype control or anti-HLA I. Supernatants were collected and percent specific lysis was calculated. Experiments were performed in triplicate. Primary NK cells were isolated from Peripheral Blood Mononuclear Cells by depletion of non-NK cells through magnetic microbead negative selection NK isolation kit (Miltenyi Biotec, Cologne, Germany). Primary NK cells were cultured in RPMI media supplemented with 15% FBS and 1000 units/mL recombinant human IL-2 (eBioscience, San Diego, CA) for one week prior to use. NKp44 expression was confirmed by flow cytometry. ^51^Cr release assay was performed with primary NK cells in the same manner as with NK92-MI cells, but at ratios of 10∶1, 5∶1, and 1∶1.

## Results

### Anti-HLA I Antibody Blocks Binding of NKp44 Fusion Protein

**Figure 1:**
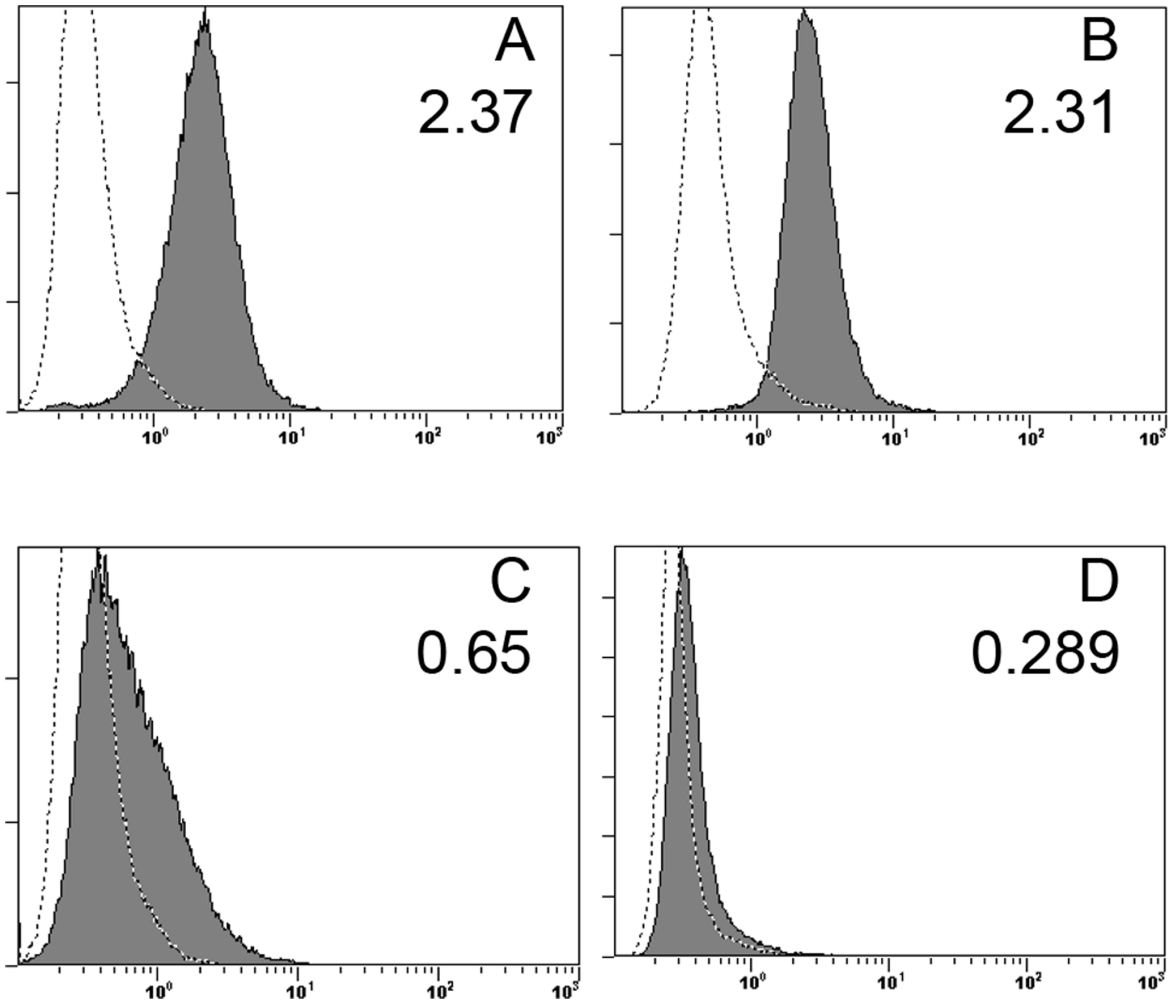
HLA I Antibody blocks binding of NKp44 fusion protein. (**A**) DB cells express a ligand for NKp44 as seen through binding of 70 µg of NKp44-Ig (1 µg\ul), detected by anti-IgG-Fc-PE (filled histogram). As a negative control, DB cells were incubated with concentrated untransfected supernatant from HEK-293 cells (empty histogram). DB cells were first blocked with Human IgG Fc fragment to prevent reverse binding of fusion protein. (**B**) DB cells were incubated with mouse IgG2a isotype control (empty histogram) or 2.5 µg anti-HLA I, detected by anti-mIgG (H+L)-PE (filled) to measure expression of cell surface HLA I. (**C**) When incubating DB cells with 2.5 µg anti-HLA I antibody prior to incubation with 70 µg of NKp44 fusion protein and staining with anti-IgG-Fc-PE (filled histogram), fusion protein binding is reduced to near background levels as seen when staining DB cells with untransfected supernatant(empty histogram). Incubating cells with mouse IgG2a isotype did not block binding of NKp44 fusion protein (data not shown). (**D**) DB cells were incubated with 2.5 µg of anti-PCNA-Alexa-488 versus mIgG2a isotype control, demonstrating uniform expression of PCNA on the extracellular surface of DB cells. Mean Fluorescent Intensity is indicated in the top right corner of all plots.

To investigate potential interactions between HLA I and NKp44 we first identified a cell line which consistently expressed a ligand for NKp44 by utilizing a soluble NKp44 fusion protein (NKp44-Ig) containing two extracellular domains of NKp44 fused to the Fc region of human IgG. Fusion protein was incubated with numerous tumor cell lines and cells were analyzed for fusion protein binding by flow cytometry. A Human Diffuse B cell Lymphoma (DB) cell line was identified which consistently expressed elevated levels of a ligand for NKp44 (Figure 1A).

**Figure 2:**
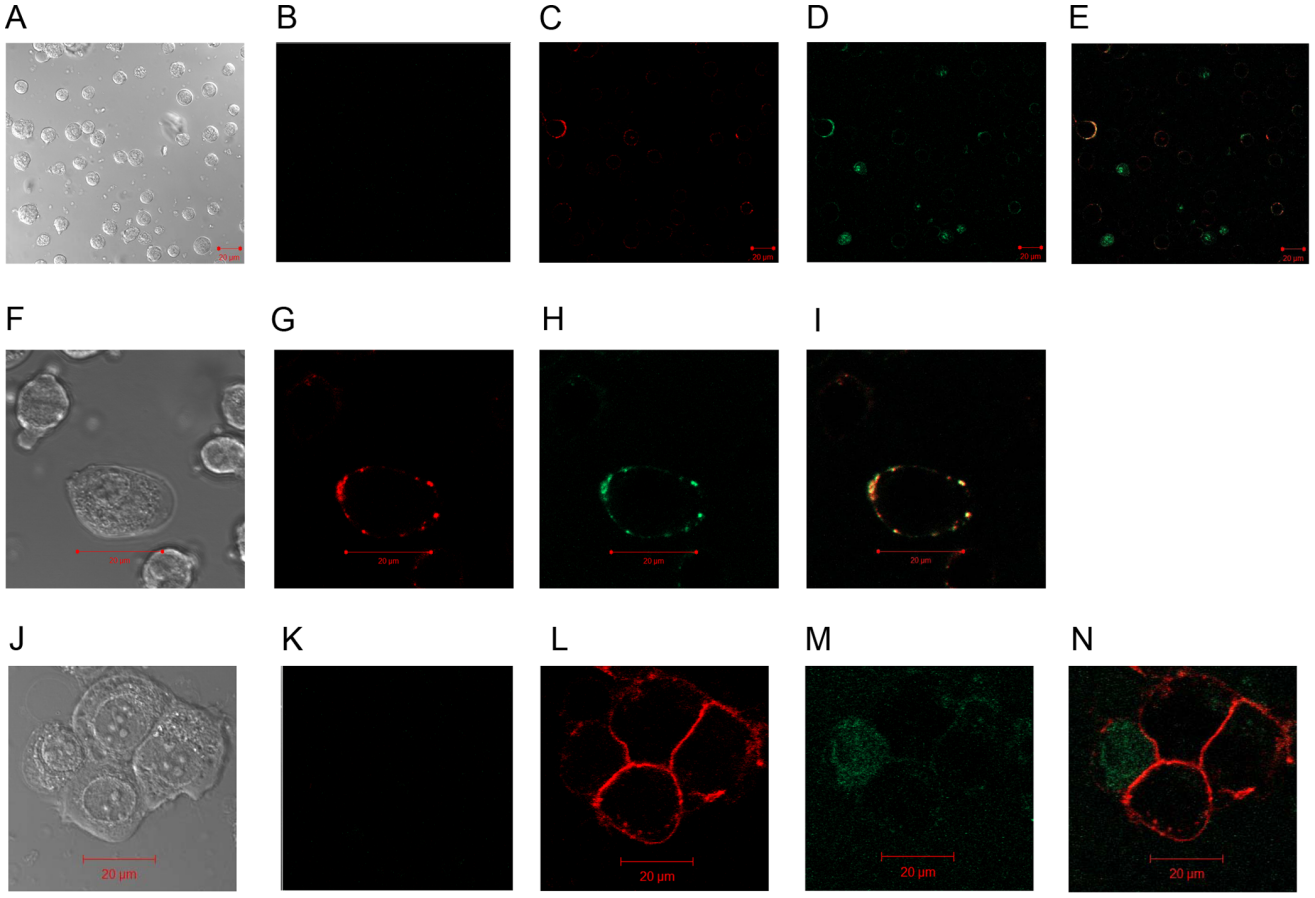
PCNA colocalizes with HLA I on the cell surface. DB and MCF-7 cells were first incubated with 2.5 µg anti-HLA I followed by staining with anti-mIgG-Dylight 594 (Red). Cells were then incubated with 2.5 µg anti-PCNA-Alexa Fluor 488 (Green). Cells were imaged on the Zeiss LSM 510 Confocal Laser Microscope using 40×, 1.2 NA, 0.28 WD (water), C-Apochromat objective utilizing 561 nm and 488 nm wavelengths at 43% and 57% transmittance respectively. Areas of colocalization can be seen as yellow when overlaid. (**A**) DB morphology. (**B**) DB cells were incubated with mIgG2a-Dylight 594 and mIgG2a-Alexa Fluor 488 and overlaid for background control. (**C**) HLA I expression on DB cells. (**D**) PCNA expression on DB cells. (**E**) Colocalization (Yellow) of HLA I and PCNA on the surface of DB cells. (**F**) Morphology of DB cell examined under 3× zoom. (**G**) HLA I expression on DB cell 3× zoom. (**H**) PCNA expression on DB cell 3× zoom. (**I**) Colocalization of HLA I and PCNA (Yellow) on DB cell 3× zoom. (**J**) Morphology of MCF 7 cells, 2× zoom. (**K**) MCF-7 cells were incubated with mIgG2a-Dylight 594 and mIgG2a-Alexa Fluor 488 and overlaid for background control. (**L**) HLA I expression on MCF-7 cells, 2× zoom. (**M**) PCNA expression on MCF-7 cells, 2× zoom. (**N**) HLA I and PCNA do not colocalize on the surface of MCF-7 cells, 2× zoom.

We then sought to block binding of the NKp44 fusion protein to DB cells utilizing a pan HLA I antibody, W6/32, which binds HLA-A, B, C, and also non-classical HLA-E. First, HLA I expression by DB cells was confirmed by flow cytometry (Figure 1B). Incubating DB cells with 2.5 µg of anti-HLA I antibody fully saturated the cells as increasing amounts did not result in any further shift of peak fluorescence away from isotype control staining. To then test if NKp44 may be interacting with HLA I, cells were first incubated with 2.5 µg of unconjugated W6/32 antibody. After washing, cells were then incubated with 70 µg NKp44 fusion protein and analyzed for fusion protein binding (Figure 2C). Prior incubation with W6/32 antibody eliminated binding of NKp44 fusion protein to near background levels, indicating interactions between NKp44 and HLA I. The epitope recognized by W6/32 is comprised of residues from all three heavy chain domains of HLA I and β-2-microglobulin, which are crucial for proper folding of the protein and presentation of antigen [28]. Thus, NKp44 interacts with fully assembled HLA I on the cell surface, and not open conformers of HLA I which do not contain β-2-microglobulin.

### PCNA Colocalizes with HLA I on the Cell Surface

Since PCNA was recently identified as a ligand for NKp44, we next sought to visualize interactions between PCNA and HLA I on the surface of live DB cells by employing confocal microscopy. We first confirmed DB cells uniformly express PCNA on the cell surface by flow cytometry in Figure 1D. Flow cytometry analysis indicates a single population of DB cells which express PCNA on the cell surface. For confocal imaging, DB cells were first stained with 2.5 µg of W6/32 anti-HLA I antibody followed by detection with anti-mIgG2a-Dylight 594. Cells were then stained with anti-PCNA-Alexa Fluor 488 and examined under 40× magnification. As seen in panels D and H of Figure 2, PCNA is localized only to the extracellular membrane as cells were alive when imaged and not permeabilized during staining. Additionally, PCNA is concentrated in areas which colocalize with dense pockets of HLA I on the surface of numerous cells (Figure 2 E and I). MCF-7 breast cancer cells were also imaged to determine if PCNA and HLA I colocalization was a common phenomenon, or unique to DB cells. While MCF-7 cells express ample HLA I on the cell surface (Figure 2L), PCNA is not uniformly expressed on the cellular surface (Figure 2M), nor did it colocalize with HLA I (Figure 2N). This data conclusively demonstrates PCNA is localized to the cell surface without external stimulus and colocalizes with HLA I on the surface of DB cells.

**Figure 3:**
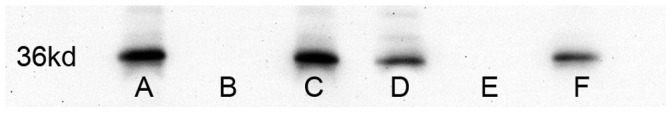
Coimmunoprecipitation of PCNA with HLA I. DB and DU145 cell lysates were first cleared of endogenous and cancerous immunoglobulins, as detailed in material and methods prior to precipitation experiments. Precleared lysates from DB (lanes **A**–**C**) and DU145 (lanes **D**–**F**) cells were subjected to immunoprecipitation with 3 µg of anti-β-2-microglobulin antibody (lanes **A, D)** or mouse IgM control of irrelevant specificity (lanes **B, E**). Precleared cell lysate was used as a positive control (lanes **C**–**F**). Reduced samples were resolved on a 10% SDS-PAGE gel and blotted with anti-PCNA antibody.

To further confirm PCNA and HLA I interactions, DB cell lysate was subjected to immunoprecipitation with anti-β-2-microglobulin or control mIgM antibody of irrelevant specificity. W6/32 antibody could not be used in this experiment since it is the same isotype (mIgG2a) as PC-10, anti-PCNA antibody, used to probe immunoprecipitation elutions. Additionally, DB cells express endogenous immunoglobulins and DU145 cells are reported to express cancerous immunoglobulins [27]. Thus, cell lysate was cleared of immunoglobulins prior to immunoprecipitation with anti-β-2-microglobulin, as detailed in materials and methods, to eliminate any potential interference. Aliquots of native elutions were reduced with 1% DTT and heated to 50°C for one hour to dissociate possible membrane protein aggregates. After resolution on a 10% SDS-PAGE gel and protein transfer, membranes were probed with anti-PCNA antibody and detected with anti-mIgG2a-HRP. PCNA was detected in elutions from DB cell immunoprecipitations performed with anti-β-2-microglobulin (Figure 3, lane A), and in precleared lysate used as a positive control (Figure 3, lanes C). However, control mIgM antibody (Figure 3, lane B) did not precipitate PCNA. Coimmunoprecipitation of PCNA with anti-β-2-microglobulin antibody was also confirmed in DU145 prostate cancer cells (Figure 3, lanes D–F).

### PCNA/HLA I Complex Interaction with NKp44 Inhibits NK Cell Cytotoxic Function

**Figure 4:**
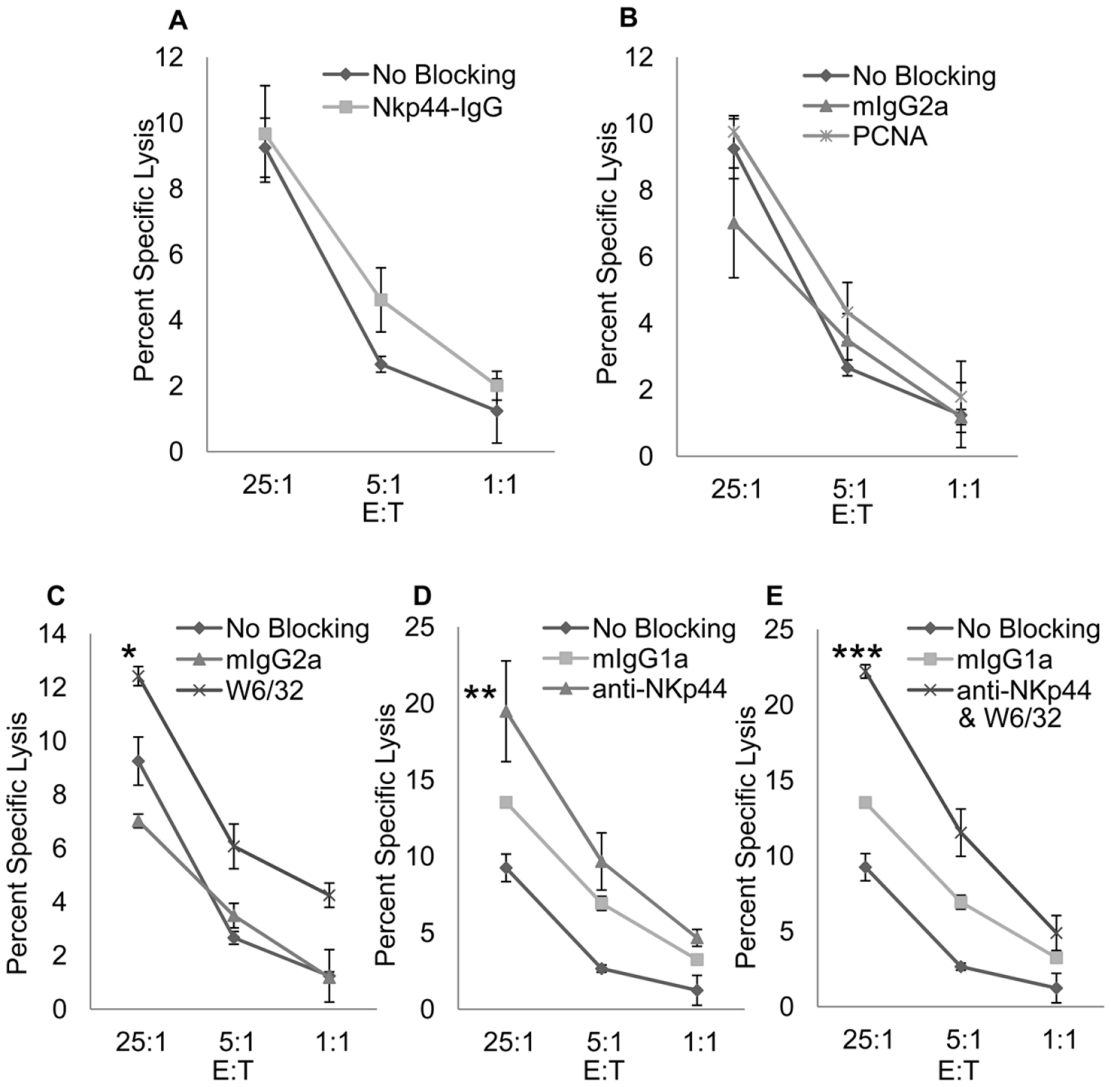
PCNA/HLA I complex interaction with NKp44 inhibits NK92-MI Cell Cytotoxic Function. The lysis of DB cells by NK92-MI cells was determined by a standard ^51^Cr release assay. DB cells and NK92-MI cells were first blocked with Human IgG Fc fragment to prevent reverse binding of fusion protein and antibody dependent cellular cytotoxicity. DB cells were loaded with ^51^Cr and incubated with 1 µg/ul of NKp44-Ig (**4A**) or 0.5mg/ml anti-PCNA (**4B**) or anti-HLA I (**4C**). DB cells were then incubated with NK92-MI cells at varying target to effector cell ratios for 4 hours at 37°C. Level of killing was compared to DB cells incubated with 0.5 mg/ml mIgG2a isotype antibody or no antibody, which served as a positive control (No Blocking) of cell lysis under unblocked conditions. Alternatively, NK92-MI cells were incubated with 0.5 mg/ml anti-NKp44 or mIgG1 isotype control antibody prior to incubation with DB cells incubated with no antibody (**4D**) or 0.5 mg/ml anti-HLA I (**4E**). Figure is representative of two independent experiments performed in triplicate. Bars ± SD. *p<.05, **p<.01, ***p<.0001, ANOVA.

NKp44 was originally described as an activating receptor when first discovered over ten years ago. However, recent evidence demonstrates the dual nature of NKp44 signaling with ITAMs in DAP-12 facilitating activating signaling and a putative ITIM sequence in the cytoplasmic tail of NKp44 facilitating inhibitory signaling [7], [8], [10], [11]. Therefore, we sought to investigate the influence PCNA colocalization with HLA I may have on cytotoxic function of NKp44 expressing NK cells. DB cells were incubated with NKp44-Ig to block interactions with NK92-MI cells via the PCNA/HLA I NKp44 ligand complex as a whole. DB cells were also incubated with anti-PCNA or anti-HLA I antibodies to individually block PCNA or HLA I interactions between DB cells and NK92-MI cells. As expected, blocking interactions with the NKp44 ligand, HLA I, or PCNA increased specific lysis of DB cells (Figure 4A–C), indicating NKp44 is indeed acting in an inhibitory fashion when interacting with HLA I/PCNA complexes. Furthermore, we blocked the NKp44 receptor on NK92-MI cells either alone (Figure 4D), or in conjunction with blocking HLA I on target cells (Figure 4E). Blocking NKp44 increased killing of DB cells and this killing was significantly increased with cooperative blocking of HLA I (Figure 4E).

**Figure 5:**
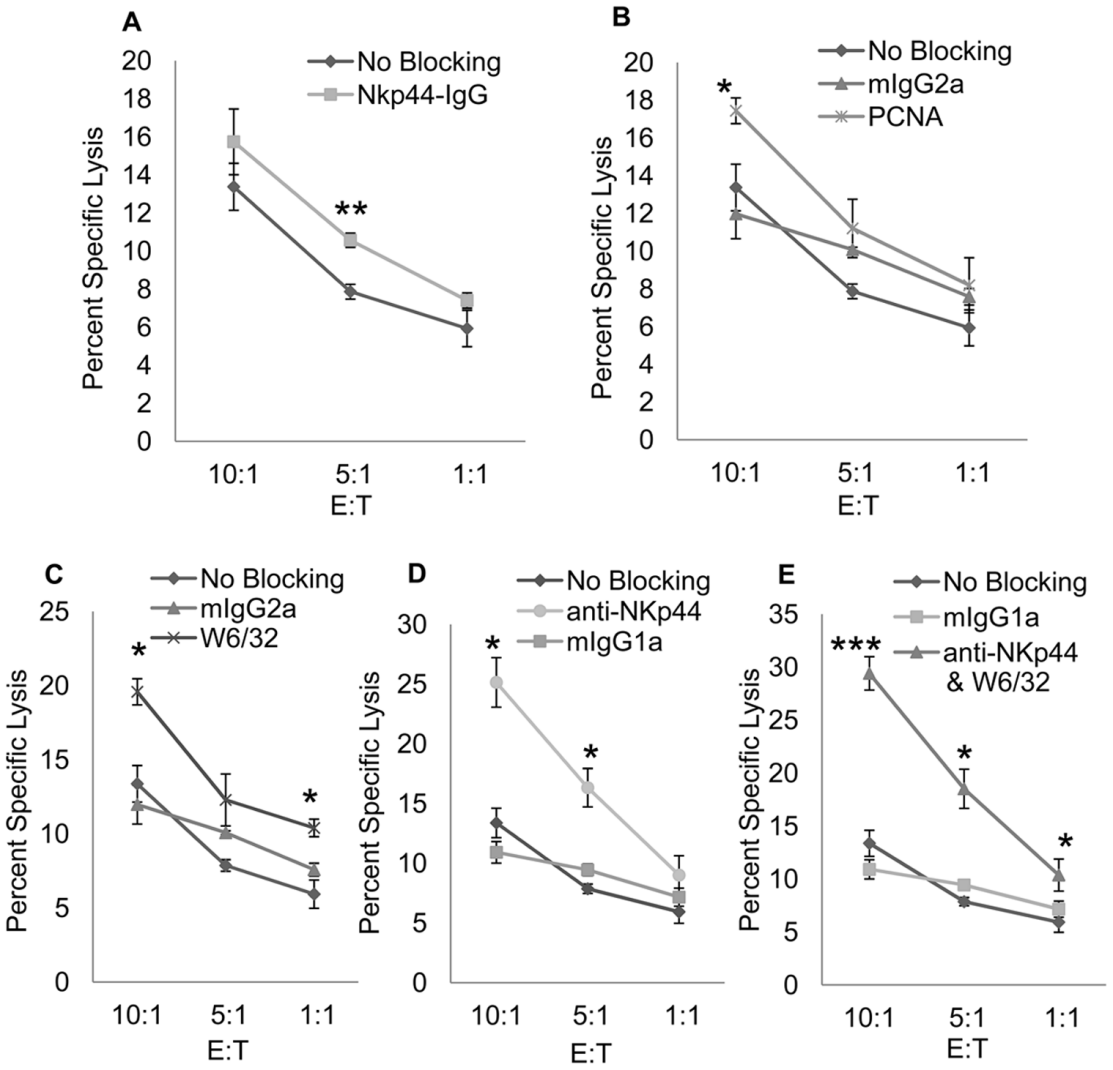
PCNA/HLA I complex interaction with NKp44 inhibits Primary NK Cell Cytotoxic Function. In order to confirm the results in figure 4, we performed a ^51^Cr release assay with primary NK cells isolated from Peripheral Blood Mononuclear Cells. Primary NK cells were cultured in 1000 units/ml recombinant human IL-2 for one week, at which point NKp44 expression was confirmed prior to use. DB cells were loaded with ^51^Cr and incubated with 1 µg/ul of NKp44-Ig to block the entire NKp44 ligand complex (**5A**) or 0.5 mg/ml anti-PCNA (**5B**) or anti-HLA I (**5C**) to individually block PCNA or HLA I interaction with NKp44. DB cells were then incubated with primary NK cells at 10∶1, 5∶1, and 1∶1 effector to target cell ratios for 4 hours at 37°C. Level of killing was compared to DB cells incubated with 0.5 mg/ml mIgG2a isotype antibody or no antibody (No Blocking). In figure 5D, NKp44 was blocked on primary NK cells with 0.5 mg/ml anti-NKp44 or mIgG1 isotype control antibody prior to incubation with DB cells incubated with no antibody. NKp44 on primary NK cells was again blocked with 0.5 mg/ml of anti-NKp44 and incubated with DB cells incubated with 0.5 mg/ml anti-HLA I in figure 5E. Bars ± SD. *p<.05, **p<.01, ***p<.0005, ANOVA.

In order to determine whether PCNA/HLA I complex interaction with NKp44 inhibits human primary NK Cell cytotoxic function, we isolated primary NK cells from Peripheral Blood Mononuclear Cells (PBMC) of healthy individuals. The primary NK cells were cultured for one week in recombinant human IL-2 and used as effector cells. The experiments were conducted under the same specifications as for NK92-MI cells. As seen in Figure 5, blocking PCNA/HLA I complex interaction with NKp44 inhibited the cytolytic function of primary NK cells. While the NKp44 antibody increased killing presumably through functional blocking of NKp44 inhibition, this antibody may also have stimulatory capacities by cross-linking the receptor, which may also yield increased killing. However, previous reports indicate PCNA recognition results in inhibition of NK cell cytotoxicity via NKp44 and our results corroborate this outcome. Moreover, our results show the complete picture of inhibitory NKp44 recognition of target cells, underlying the role of HLA I and PCNA interaction. However, it will be interesting to know whether PCNA itself is inhibitory, or its association with HLA I is responsible for inhibition.

## Discussion

NK cells represent a special population of lymphoid cells which are amongst the first line of defenders against microbial or virally infected cells as well as tumor formation. NK cells are able to survey the body and discern the health of cells and tissue through surface proteins interacting with activating and inhibitory receptors on the NK cell surface. Of these receptors, the NCRs are vital in activating NK cells and evoking powerful effector functions in the way of cellular cytotoxicity and cytokine secretion. Due to the extreme deadliness of NK cytotoxicity, effector functions are highly regulated by the balance of signaling from inhibitory and activating receptors binding respective ligands on the surface of target cells. It is in the later stages of this fine balance where signals transduced through NKp44 influence NK cell functions by conveying either an activating signal through recognition of an unknown ligand, or an inhibitory signal through recognition of PCNA [7], [8].

In this report we have identified interaction between NKp44 on NK cells and HLA I in complex with PCNA on target cells. We demonstrate that DB cells uniformly express PCNA on the cell surface and Class I HLA antibody, W6/32, blocks binding of NKp44-Ig fusion protein to the surface of DB cells (Figure 1). We further show HLA I and PCNA colocalize on the surface of DB cells as visualized through confocal microscopy (Figure 2). This association was confirmed in DB cells as well as DU145 prostate cancer cells through coimmunoprecipitation (Figure 3). Finally, we show HLA I in association with PCNA participates in induction of inhibitory signaling through NKp44 on NK92-MI cells (Figure 4) and primary NK cells (Figure 5).

Rosental *et al.* recently reported PCNA expression on the surface of cancer cells, but only in the presence of NKp44 expressing NK cells or over expression of PCNA through transfection [8]. In this report, we find PCNA is constitutively expressed on the cell surface of DB cells without gene transfection or the presence of NK cells. We propose several key aspects that may account for this difference. First, Rosental *et al.* utilized cell lines of epithelial origin to demonstrate their results [8]. We have utilized a B cell lymphoma cell line of hematopoietic/immunologic origin. DB cells used in this report differ greatly from the epithelial cells used in Rosental *et al.* DB cells arise from a completely separate line of progenitor cells, thus executing different genetic expression programs as compared to cells of epithelial origin. DB cells also grow in large globular clumps of cells in suspension, thus exhibiting completely different morphology and growth kinetics than epithelial cells, which grow as a flattened single cell layer in tissue culture. Furthermore, we have confirmed in Figure 2 that MCF-7 cells grown on a cover slip do not express PCNA on the cell surface. We have also seen this result in DU145 cells grown on a cover slip (data not shown). As seen in Figure 2, DB cells do express PCNA on the cell surface. Surface PCNA expression in DB cells was confirmed by flow cytometry, which demonstrated one uniform population, indicating all DB cells in culture express PCNA on their cell surface (Figure 1D). As DB cells replicate very quickly, endogenous PCNA levels are likely higher than other slower replicating cell lines, which may account for the steady expression of PCNA on the surface of DB cells. Additionally, PCNA expression is also dependent on p53 status of the cell. Wild type p53 specifically trans-activates the PCNA promoter at p53 response elements when p53 levels are low and represses PCNA expression at high levels of p53 during cellular stress, preventing replication [29]. However, mutant p53 nonspecifically trans-activates the PCNA promoter, resulting in rampant expression of PCNA in malignant cells [30]. In this light, cell surface PCNA may be an indicator of overall cell health, p53 status, and a marker for early onset of cancer transformation. Previous reports have also indicated Bat3, which controls acetylation and thus activation of p53, also colocalizes with HLA I on the surface of tumor and dendritic cells, where it serves as a ligand for NKp30 [20], [21], [31]. This suggests NCR ligands are a complex involving HLA I and DAMP molecules, which bind or colocalize to HLA I forming a complex of molecules enabling recognition by the NCRs. Since NKp30 recognizes Bat3 and NKp44 recognizes PCNA, the NCRs may recognize a multitude of different DAMP molecules, leading to diverse activation or inhibition of NK effector functions.

Modulation of NK cell activity may not only depend on the DAMP molecule associated with HLA I, but also the NCR that is recognizing the motif. Due to the dual nature of NKp44 signaling, it will be of interest to determine if recognition of PCNA, HLA I, or the motif as a whole is responsible for inhibition of NK cytotoxicity. Neither NKp30 nor NKp46 has been reported to contain an ITIM sequence; however, an immunosuppressive isoform of NKp30 resulting from a single-nucleotide polymorphism in the 3′ untranslatable region has been reported [32]. Thus, NCRs may exhibit both inhibitory and activating functions. Whether the divergence of NKp44 functions depends on the individual DAMP molecule associated with HLA I or solely the presence of HLA I, implicating a unique activating ligand, remains to be elucidated. Interestingly, DAMP molecules High-Mobility Group Protein B1 and S100A8/9 have the ability to bind heparin sulfate and heparin sulfate proteoglycans, which are known to be coligands involved in NCR dependent recognition of tumor cells, resulting in secretion of IFN-γ but not cytotoxicity [33]–[35]. We postulate a DAMP molecule may be the missing link in heparin sulfate dependent recognition of tumor cells, which would then elicit full NK cell effector function.

These results also bring further attention and new function to DAMP molecules, or proteins which are located and function intracellularly, but somehow localize to the extracellular membrane, despite lacking a traditional secretory leader sequence [36]. These proteins are released by cells which have become injured in the absence of infection due to ischemia, hypoxia, transformation, chemotherapy, or other trauma [35]. Aspects of PCNA transport are still a mystery as the protein does not contain a secretory sequence or a nuclear localization signal, even though the majority is located inside the nucleus [16]. In regards to PCNA transport in DB cells, our attempts to inhibit cell surface PCNA were unsuccessful using Brefeldin A, indicating PCNA is not transported via the Golgi apparatus. Unconventional protein transport to the cell surface can occur however, by either direct transport from the cytoplasm across the plasma membrane, lysosomal secretion, exosome derived bodies, or vesicle shedding [37].

Analogous to Toll Like Receptors recognizing pathogen-associated molecular patterns, the NCRs may represent a class of receptors that participate in pattern recognition of DAMP molecules, whose identities may reflect the intracellular health of a cell in addition to the traditional method of HLA I presenting self peptide. In this manner, HLA I may also present DAMP molecules for identification by the NCRs, which would then regulate NK cell function potentially dependent on the DAMP molecule present and the NCR engaged. Knowledge of the identities and nature of DAMP molecules that bind to HLA I or other cell surface molecules to form ligands for the NCRs will shed light on NK cell recognition of target cells under healthy and disease conditions. This study further highlights the elegant complexity of NK cell recognition and activation by target cells.

### Conclusions

In this study, we have identified interactions between PCNA and HLA I on the extracellular surface of tumor cells, which forms a complex ligand for NKp44. We also demonstrated that PCNA localization to the extracellular surface does not absolutely require the presence of an NKp44 expressing NK cell or over expression due to gene transfection. Interaction between NKp44 and PCNA in complex with HLA I resulted in inhibition of NK cell cytotoxicity.
